# Olfactory Dysfunction and Sinonasal Symptomatology in COVID-19: Prevalence, Severity, Timing, and Associated Characteristics

**DOI:** 10.1177/0194599820929185

**Published:** 2020-05-19

**Authors:** Marlene M. Speth, Thirza Singer-Cornelius, Michael Oberle, Isabelle Gengler, Steffi J. Brockmeier, Ahmad R. Sedaghat

**Affiliations:** 1 Klinik für Hals-, Nasen-, Ohren- Krankheiten, Hals-und Gesichtschirurgie, Kantonsspital Aarau, Aarau, Switzerland; 2 Institute for Laboratory Medicine, Kantonsspital Aarau, Aarau, Switzerland; 3 Department of Otolaryngology–Head and Neck Surgery, University of Cincinnati College of Medicine, Cincinnati, Ohio, USA

**Keywords:** coronavirus, COVID-19, SARS-CoV2, SARS-CoV-2, anosmia, hyposmia, olfactory dysfunction, olfactory function, gustatory dysfunction, gustatory function, olfaction, smell, taste, nasal obstruction, rhinorrhea

## Abstract

**Objective:**

Olfactory dysfunction (OD)—hyposmia or anosmia—is a symptom of coronavirus disease 2019 (COVID-19), caused by the novel coronavirus, severe acute respiratory syndrome coronavirus 2 (SARS-CoV-2). We sought to better determine prevalence, severity, and timing of OD in COVID-19 relative to other sinonasal and pulmonary symptoms.

**Study Design:**

Prospective, cross-sectional.

**Setting:**

Regional/cantonal hospital.

**Subjects:**

In total, 103 patients diagnosed with COVID-19 with reverse transcription polymerase chain reaction (RT-PCR)–based testing.

**Methods:**

All patients testing positive for COVID-19 at Kantonsspital Aarau over a 6-week period were approached. Timing and severity (at its worst, on scale of 0 [none], 1 [mild], 2 [moderate], and 3 [severe]) of OD, loss of taste, nasal obstruction, rhinorrhea/mucus production, fever, cough and shortness of breath (SOB) were assessed for each patient.

**Results:**

Prevalence of OD was 61.2%, and severity of OD was strongly correlated with severity of loss of taste experienced (ρ = 0.87, *P* < .001). OD was experienced on the first day of COVID-19 by 8.7% and overall occurred at median infection day 3 (mean, 3.4; range, 0-12). Most experiencing OD reported anosmia, and mean severity of all with OD was moderate to severe (mean [SD], 2.7 [0.6]). Nasal obstruction (49.5%) and rhinorrhea (35.0%) were frequently reported but not correlated with OD. SOB was more severe in patients with OD. OD was associated negatively with older age (OR, 0.96; 95% CI, 0.93-0.99; *P* = .007) and positively with female sex (OR, 2.46; 95% CI, 0.98-6.19; *P* = .056).

**Conclusions:**

OD is highly prevalent during COVID-19, occurring early and severely, often in conjunction with loss of taste. OD is associated negatively with older age and positively with female sex. Patients with OD may also experience more severe SOB.

Coronavirus disease 2019 (COVID-19), which is caused by the severe acute respiratory syndrome coronavirus 2 (SARS-CoV-2) virus, first presented in December 2019 in Wuhan, China, and has quickly spread all around the world.^1,2^ Characterized by the World Health Organization as a pandemic and global health emergency on March 11, 2020,^3^ SARS-CoV-2 has infected millions of individuals and killed hundreds of thousands as we write. COVID-19 is most frequently characterized by symptoms of fever, cough, and shortness of breath as well as constitutional symptoms such as fatigue and myalgias.^2,4,5^ By contrast, although the nasal cavity plays a prominent role in COVID-19—the nasal cavity likely is the site of viral entry and is also the seat of vigorous viral reproduction^6^—initial reports of nasal symptoms in COVID-19 have suggested low prevalence, experienced by less than 10% of infected patients.^7,8^

As the number of COVID-19 cases increased around the world, it has become apparent that sudden-onset olfactory dysfunction (OD)—hyposmia or anosmia—may be indicative of COVID-19.^9,10^ The prevalence of OD among COVID-19 patients has been reported to be as high as 85.6% and almost uniformly associated with concomitant subjective gustatory dysfunction/loss of taste.^11^ Another recent study of 237 COVID-19 patients experiencing OD from the American Academy of Otolaryngology–Head and Neck Surgery COVID-19 Anosmia Reporting Tool reported that OD could occur at any time during the course of infection but usually began early in the COVID-19 disease course.^12^ The objective confirmation of OD in patients testing positive for COVID-19 has been reported by Moein et al,^13^ who used the University of Pennsylvania Smell Identification Test to find 98% of a COVID-19 cohort had hyposmia or anosmia.

OD therefore appears to be a highly prevalent symptom of COVID-19, and sudden-onset OD should be considered a potential predictor of COVID-19. At present, the time course and severity of OD as well as its association with sinonasal or other symptoms experienced by COVID-19 patients remain incompletely characterized. The objective of our study was to characterize the prevalence, timing, and the severity of patient-reported OD, as well as other sinonasal symptoms and their association with the classic symptoms of COVID-19, such as fever, cough, and shortness of breath (SOB). In doing so, we hope to shed more light on the pervasiveness of OD and sinonasal symptomatology in COVID-19 and their significance as pathognomonic symptoms of the disease.

## Methods

### Study Participants

This study was approved by the institutional review board of the Kantonsspital Aarau (Ethikkomission Nordwest und Zentralschweiz) in Aarau, Switzerland. Patients, receiving their care at the Kantonsspital Aarau who tested positive for COVID-19 at this cantonal hospital between March 3, 2020, and April 17, 2020, were identified and contacted. All patients had been tested for COVID-19 using a reverse transcription polymerase chain reaction (RT-PCR)–based test. All patients who participated provided consent to participate in this study. All patients were then contacted by telephone up to 3 times to complete the study. Patients who were not reachable with 3 telephone calls were excluded. Patients who were hospitalized were also approached in person. Patient who were in intensive care units or who were deceased were excluded.

### Study Design

This was a prospective, cross-sectional telephone questionnaire study of patients diagnosed with COVID-19 at the Kantonsspital Aarau. Demographic characteristics of the participants—age, sex, smoking history, and histories of allergic rhinitis/hay fever, chronic rhinosinusitis, and asthma—were collected.

A standardized questionnaire was given to participants. Participants were asked how many days they had been experiencing symptoms of COVID-19 and also how many days into the COVID-19 course that they began to experience OD, specifically. Then they were asked to provide a qualitative assessment of their clinical signs and the exact order in which they had experienced the symptoms. Participants were asked to rate their sense of smell and sense of taste, each at its worst point during the infection compared to baseline, as “normal,”“decreased,” or “none at all.” For participants reporting “decreased” or “none at all,” a follow-up question was provided asking how many days after the onset of COVID-19 symptoms any decreased senses of smell and taste began.

All participants were then specifically asked about the symptoms of decreased sense of smell, decreased sense of taste, nasal obstruction, rhinorrhea/nasal mucus production, fever, cough, and shortness of breath. For each of these symptoms, patients were asked to rate the severity of the symptoms, at its worst during the COVID-19 course, on a scale of 0 (none), 1 (mild), 2 (moderate), or 3 (severe)—a scale that was modeled on the validated nasal symptom score.^14^

### Statistical Analysis

All analysis was performed with the statistical software package R (www.r-project.org). Although we offered inclusion of all eligible patients not meeting exclusion criteria, our goal was to include at least 100 participants based on a sample size calculation for prevalence of OD (hyposmia or anosmia) with a conservative a priori population prevalence assumption of 0.5 and with marginal error = 0.1.^15^ Basic, standard descriptive statistics were performed. A 95% confidence interval for the presence of binomially distributed variables (eg, the prevalence of loss of smell) was calculated using Wilson’s method. Correlation was performed using Spearman’s method. Logistic regression was used to identify factors associated with experiencing OD. In the multivariable analysis, significant predictors were identified via backward elimination, using a *P* value cutoff of 0.100. The final multivariable results were cross-validated by bootstrapping the data over 100 iterations. For each variable retained in the final model, a *P* value and a log-odds ratio were calculated.

## Results

### Characteristics of Study Participants

**Figure 1.**
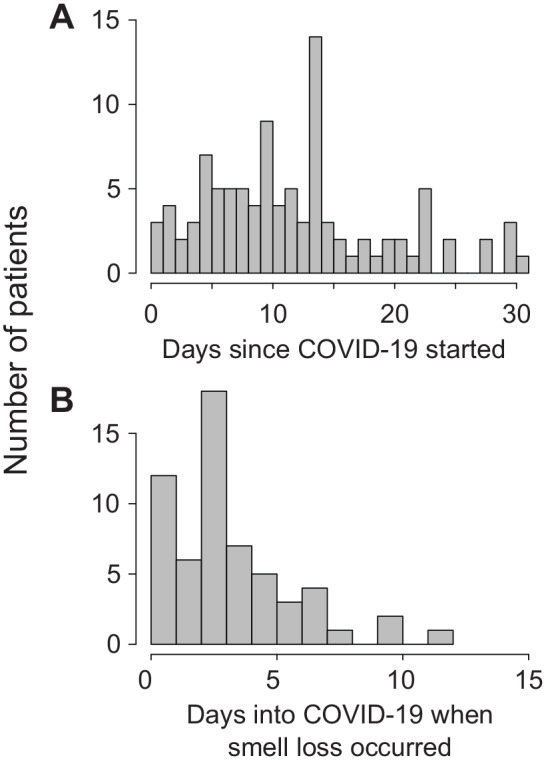
Histogram plots of (A) how long ago participants began experiencing coronavirus disease 2019 (COVID-19) symptoms and (B) how many days into COVID-19 did smell loss begin.

A total of 103 participants (48.5% male, 51.5% female) were recruited, and their characteristics, including percentage of participants experiencing various symptoms of COVID-19, are listed in **Table 1**. Of all participants, 4 were hospitalized as inpatients and 19 had been previously hospitalized for COVID-19 but had since been released. At the time of the interview, 3 participants could not remember or identify when their COVID-19 symptoms started, but the other 100 participants reported that their COVID-19 symptoms had started a median of 11 days (mean, 12.3 days; range, 0-31 days) ago (**Figure 1A**). The prevalence of patient-reported inflammatory airway conditions was 35% for allergic rhinitis, 1% for chronic rhinosinusitis, and 12.6% for asthma.

**Table 1.** Characteristics of Study Participants (N = 103).

Characteristic	Value
Demographics	
Age, mean (SD), y	46.8 (15.9)
Sex, %	
Male	48.5
Female	51.5
Smoking history, %	
Never smoker	72.8
Former smoker	18.4
Current smoker	8.8
Comorbidities, %	
Allergic rhinitis or hay fever	35.0
Chronic rhinosinusitis or polyps	1.0
Asthma	12.6
COVID-19 symptom characteristic	
Days since symptoms started, mean (SD)	12 (7)
Symptoms experienced, %	
Olfactory dysfunction	61.2
Gustatory dysfunction	65.0
Nasal obstruction	49.5
Mucus production	35.0
Fever	74.8
Cough	68.0
Shortness of breath	46.6

Abbreviation: COVID-19, coronavirus disease 2019.

### Prevalence, Severity, and Timing of Olfactory and Gustatory Dysfunction in COVID-19 Patients

**Figure 2.**
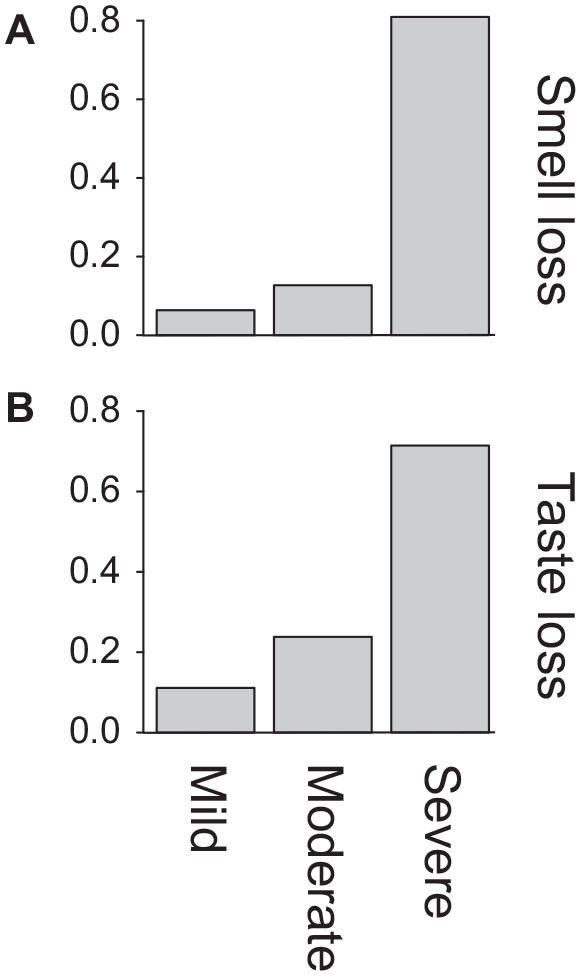
Bar plots showing the fraction of patients reporting mild, moderate, or severe decrease in sense of (A) smell and (B) taste in patients reporting some decrease in those senses, respectively.

Of the 103 participants, 14.6% (95% CI, 9.0%-22.6%) reported that their sense of smell was “decreased” and 46.6% (95% CI, 37.3%-56.2%) reported that their sense of smell was “none at all” at its worst during the course of the disease and when compared to baseline. The prevalence of OD in our cohort was therefore 61.2% (95% CI, 51.5%-70.0%). OD occurred on the first day of COVID-19 for 8.7% of our participants. OD with no other symptoms occurred on the first day of COVID-19 in 2.9% of the cohort. For participants who experienced OD, the OD occurred on the first day in 15.3% (in 5.1% on the first day without any other symptoms). Relative to the beginning of COVID-19 symptoms, OD began at a median time of 3 days (mean, 3.4 days; range, 0-12 days) (**Figure 1B**). Of the patients who reported some OD, 6.3% stated that the severity was “mild,” 12.7% reported that it was “moderate,” and 81.0% reported that it was “severe” at its worst during the COVID-19 course (**Figure 2A**).

Similar findings were identified for participants’ sense of taste. At its worst during the COVID-19 course, 25.2% (95% CI, 17.8.3%-34.4%) stated that their sense of taste was “decreased” and 39.8% (95% CI, 30.9%-49.5.4%) reported that their sense of taste was “none at all.” Thus, 65.0% (95% CI, 55.5%-73.6%) reported having at least some decrease in sense of taste during the COVID-19 course. Of the patients who reported some loss of their sense of taste, 10.4% stated that the severity was “mild,” 22.4% reported that it was “moderate,” and 67.2% reported that it was “severe” at its worst during the COVID-19 course (**Figure 2B**).

We next checked for correlation between OD and loss of sense of taste. Among the entire study cohort, ratings of patients’ sense of smell and sense of taste on the scale of “normal,”“decreased,” or “none at all” were strongly correlated (ρ = 0.86; 95% CI, 0.78-0.92; *P* < .001). Similarly, the severities of OD and decrease in sense of taste rated as “none,”“mild,”“moderate,” or “severe” were highly correlated (ρ = 0.87; 95% CI, 0.79-0.93; *P* < .001).

### Prevalence and Severity of sinonasal symptomatology, fever, cough, and Shortness of Breath

**Figure 3.**
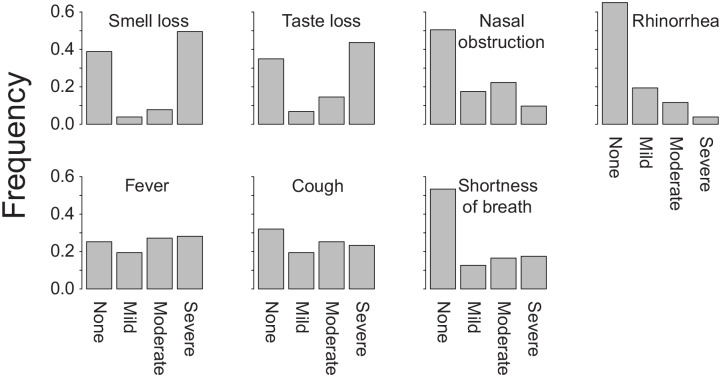
Bar plots showing fraction of patients reporting mild, moderate, or severe symptoms of decreased sense of smell, decreased sense of taste, nasal obstruction, rhinorrhea, fever, cough, and shortness of breath.

For the overall cohort, **Figure 3** shows the severity ratings of decreased sense of smell, decreased sense of taste, nasal obstruction, rhinorrhea/nasal mucus production, fever, cough, and shortness of breath reported by participants at their worst during the COVID-19 course. Of the 103 participants, 49.5% reported at least mild nasal obstruction, 35.0% reported at least mild rhinorrhea/nasal mucus production, 74.8% reported at least mild fever, 68.0% reported at least mild cough, and 46.6% reported at least mild shortness of breath. The mean scores for severity ratings of decreased sense of smell, decreased sense of taste, nasal obstruction, runny nose/nasal mucus production, fever, cough, and shortness of breath are shown in **Table 2**.

**Table 2.** Coronavirus Disease 2019 Symptom Severity Ratings.

Symptom severity	All (N = 103), mean (SD)	Olfactory dysfunction (n = 63), mean (SD)	No olfactory dysfunction (n = 40), mean (SD)	*P* value^a^
Decreased sense of smell	1.7 (1.4)	2.7 (0.6)	0 (0)	<.001
Decreased sense of taste	1.7 (1.3)	2.5 (0.8)	0.4 (0.9)	<.001
Nasal obstruction	0.9 (1.1)	1.0 (1.1)	0.7 (1.0)	.172
Runny nose/nasal mucus production	0.5 (0.8)	0.6 (0.9)	0.5 (0.8)	.908
Fever	1.6 (1.2)	1.7 (1.1)	1.4 (1.2)	.149
Cough	1.4 (1.2)	1.5 (1.1)	1.2 (1.2)	.217
Shortness of breath	1.0 (1.2)	1.2 (1.2)	0.6 (1.1)	.011

a Comparison of values from patients with olfactory dysfunction to those with no olfactory dysfunction.

### Relationship of Olfactory Dysfunction With Other Symptoms of COVID-19

We next compared symptom scores of participants with OD compared to those without OD (**Table 2**). Expectedly, decreased sense of taste was significantly more severe (*P* < .001) in patients with OD (mean [SD], 2.5 [0.8]) compared to patients without OD (mean [SD], 0.4 [0.9]). Although participants who experienced OD generally had more severe symptoms, only shortness of breath was significantly (*P* = .011) more severe in patients with OD (mean [SD], 1.2 [1.2]) compared to patients without OD (mean [SD], 0.6 [1.1]).

We next sought to understand the incidence of OD with other symptoms of COVID-19. We found that only 4.8% of patients with OD experienced no symptoms of fever, cough, or shortness of breath compared to 95.2% of patients with OD who had at least one of these symptoms. We found that 34.9% of OD patients did not experience any symptoms of nasal obstruction or nasal mucus production. Of patients with OD, 54.0% experienced nasal obstruction and 34.9% experienced nasal mucus production.

**Figure 4.**
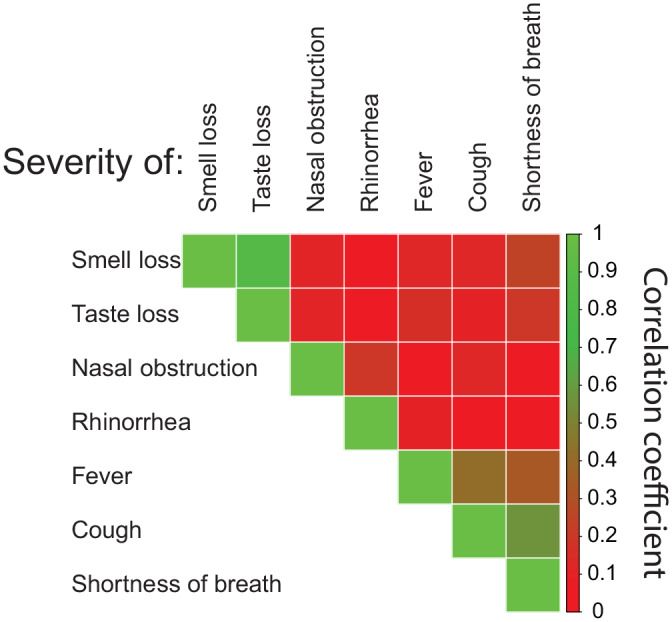
Correlation plot for severity ratings of symptoms of smell loss, taste loss, nasal obstruction, nasal mucus production, fever, cough, and shortness of breath.

Finally, we checked for correlation between the severity ratings of OD, decreased sense of taste, nasal obstruction, nasal mucus production, fever, cough and shortness of breath (**Figure 4**). As we have already described, the severity ratings of OD and decreased sense of taste were highly correlated. We also found that the severity ratings of fever, cough, and shortness of breath were correlated (**Figure 4**). There was otherwise no evidence of correlations between the symptoms.

#### Associations with olfactory dysfunction

We next sought to determine if any participant-specific characteristics or their COVID-19 course was associated with reporting OD (**Table 3**). On univariate association, we found that age (odds ratio [OR], 0.96; 95% CI, 0.93-0.99; *P* = .003) was negatively associated while female sex was positively associated (OR, 2.62; 95% CI, 1.13-6.05; *P* = .024) with reporting OD during COVID-19. These results were confirmed by multivariable analysis, which identified age to be negatively associated with OD (OR, 0.96; 95% CI, 0.93-0.99; *P* = .007) and a point estimate suggestive for positive association between female sex and OD (OR, 2.46; 95% CI, 0.98-6.19; *P* = .056).

**Table 3.** Factors Associated With Olfactory Dysfunction in COVID-19.

	Univariate analysis		Multivariable analysis	
Characteristic	Odds ratio (95% CI)	*P* value	Odds ratio (95% CI)	*P* value
Patient characteristics				
Age	0.96 (0.93-0.99)	.003	0.96 (0.93-0.99)	.007
Sex	2.62 (1.13-6.05)	.024	2.46 (0.98-6.19)	.056
Smoking	0.99 (0.65-1.51)	.978	—	.457
Allergic rhinitis	1.12 (0.48-2.62)	.801	—	.949
Asthma	0.58 (0.18-1.89)	.369	—	.186
COVID-19 symptom severities				
Nasal obstruction	1.50 (0.99-2.29)	.057	—	.278
Rhinorrhea/nasal mucus production	1.10 (0.67-1.79)	.701	—	.929
Fever	1.30 (0.91-1.86)	.153	—	.227
Cough	1.19 (0.83-1.69)	.344	—	.912
Shortness of breath	1.40 (0.97-2.03)	.076	1.43 (0.95-2.14)	.086

Abbreviations: COVID-19, coronavirus disease 2019; —, not included in the final multivariable model.

## Discussion

COVID-19 has so far infected millions and killed hundreds of thousands around the world, and it remains a global threat.^3,16,17^ Although mitigation and containment strategies have slowly begun to turn the tide of new infections, COVID-19 remains an active global pandemic, and some populous areas of the world still are in the early stages of spread.^18,19^ Moreover, even if control of COVID-19 is achieved, there is still fear of a second wave or seasonal bursts of the infection.^20^ Thus, COVID-19 remains a serious threat to health care systems and populations around the world, and more knowledge, in particular as it relates to identifying asymptomatic carriers who may be one of the primary means of spread, is necessary.^21^ When COVID-19 first came to light, symptoms of fever, cough, and shortness of breath were brought to the forefront as these symptoms were most commonly reported by patients.^2,4,5^ More recently, however, OD has been identified as a symptom of COVID-19 that may have significance in identifying asymptomatic carriers or those with mild symptoms that would otherwise not raise suspicion for COVID-19.^6,22^ Moreover, there are still few reported data on OD in the context of sinonasal or other classic symptoms of COVID-19. In our cohort of 103 COVID-19–positive patients, the prevalence of OD (hyposmia or anosmia) was 61.2% and the mean onset was 3.4 days after symptoms of COVID-19 first appeared. OD occurred on the first day of COVID-19 symptoms in 8.7% of participants and was the only symptom on the first day of symptoms in 2.9%. When it occurred during COVID-19, OD was severe in nature and was strongly correlated with a concomitant loss of taste. In contrast to prior studies reporting low prevalence of nasal symptoms, 30% to 50% of our participants experienced nasal obstruction or rhinorrhea, which they attributed to COVID-19. However, there was no correlation between these symptoms and OD. Of all patients’ characteristics as well as characteristics of the patients’ COVID-19 course, only older age was negatively associated with having OD and female sex was possibly positively associated with having OD. Interestingly, patients with OD experienced generally more severe SOB compared to patients not experiencing OD.

OD is an expected element of coronavirus pathophysiology.^6^ Expression of the SARS-CoV-2 host cell surface receptor, angiotensin-converting enzyme 2 (ACE2), is highly expressed in nasal mucosa, in particular the ciliated epithelium and goblet cells.^23,24^ Moreover, viral replication appears to be greatest in the nasal cavity, as evidenced by the highest viral titers shed from the nose.^25^ Finally, coronaviruses have been shown to be highly neurotropic in animal models where olfactory neurons have been shown to be directly permissible to infection.^26-28^

The prevalence of OD in association with COVID-19 has been reported in the literature to range from 19.4% to 85.6%.^9,11,29,30^ It has also been shown that the occurrence of OD is correlated with the occurrence of loss of taste.^11,30^ OD has been described to be the first symptom of COVID-19 in 11.8% to 27%.^11,12^ Despite rapidly expanding literature on OD in COVID-19, current knowledge gaps include the exact timing (or time distribution) of when OD occurs, the severity with which it occurs and how its severity correlates to other COVID-19 symptoms, and whether there are factors that may be associated with OD.

Our study confirms prior studies and extends them to address existing knowledge gaps. Consistent with prior findings, we observed that OD is highly prevalent in COVID-19 and frequently occurs concomitant to loss of sense of taste. We extended these findings by also showing that the severity of OD is generally quite severe, and its severity is correlated with the severity of loss of sense of taste experienced by patients. In our cohort, although the occurrence of OD as the first symptom was lower than previous reports by Lechien et al^11^ and Kaye et al,^12^ our results were consistent with theirs in finding that OD is generally an early symptom of COVID-19. We also extended these findings by specifically showing the time distribution of incidence of OD in COVID-19. In our cohort, OD occurred with a mean 3.4 days (median 3 days) into the COVID-19 course and it almost always occurred before day 8 of the disease course. We also show for the first time that 30% to 50% of patients attributed symptoms of nasal congestion and rhinorrhea to COVID-19 but that these symptoms did not correlate with OD. Classic COVID-19 symptoms of fever and cough did not correlate with OD either, although patients with OD did have more severe SOB when compared to those without OD. Finally, we identify age and sex as risk factors for OD, with younger age and female sex being associated with OD.

The results of our study should be interpreted within the constraints of its limitations. Our cohort size consisted of only 103 patients, and all were from 1 region of Switzerland. We also acknowledge that our study design relied heavily on adequate patient recall and report. However, previous studies of recall bias suggest that recall of disease-specific manifestations (such as symptoms), in particular those related to noteworthy events (such as COVID-19), is generally reliable, in particular for short periods (such as less than a month as we do here).^31^ We also studied subjective reports of OD due to present logistical constraints related to meeting with infected patients to apply objective olfactory testing. Finally, most of these patients had been experiencing symptoms for less than 2 weeks and are thus likely still in the midst of the infection. For this reason, we did not study resolution of OD, and we also acknowledge that OD could continue to evolve in those patients. Thus, it is possible, for example, that our estimates for the prevalence and timing of OD may be underestimated, respectively, compared to if queried in patients who had completely resolved infections.

## References

[1] Wu D Wu T Liu Q Yang Z. The SARS-CoV-2 outbreak: what we know. Int J Infect Dis. 2020;94:44-48. 32171952 10.1016/j.ijid.2020.03.004 PMC7102543

[2] Guan WJ Ni ZY Hu Y, et al Clinical characteristics of coronavirus disease 2019 in China. N Engl J Med. 2020;382:1708-1720. 32109013 10.1056/NEJMoa2002032 PMC7092819

[3] Mahase E. Covid-19: WHO declares pandemic because of “alarming levels” of spread, severity, and inaction. BMJ. 2020;368:m1036. 32165426 10.1136/bmj.m1036

[4] Lescure FX Bouadma L Nguyen D, et al Clinical and virological data of the first cases of COVID-19 in Europe: a case series [published online 3 27, 2020]. Lancet Infect Dis. 10.1016/S1473-3099(20)30200-0 PMC7156120 32224310

[5] Chen N Zhou M Dong X, et al Epidemiological and clinical characteristics of 99 cases of 2019 novel coronavirus pneumonia in Wuhan, China: a descriptive study. Lancet. 2020;395:507-513. 32007143 10.1016/S0140-6736(20)30211-7 PMC7135076

[6] Gengler I Wang JC Speth MM Sedaghat AR. Sinonasal pathophysiology of SARS-CoV-2 and COVID-19: a systematic review of the current evidence [published online 4 10, 2020]. Laryngoscope Invest Otolaryngol. 10.1002/lio2.384 PMC7262250 32587887

[7] Young BE Ong SWX Kalimuddin S, et al Epidemiologic features and clinical course of patients infected with SARS-CoV-2 in Singapore [published online 3 3, 2020]. JAMA. 10.1001/jama.2020.3204 PMC7054855 32125362

[8] Dong X Cao YY Lu XX, et al Eleven faces of coronavirus disease 2019 [published online March 20, 2020]. Allergy. 10.1111/all.14289 PMC7228397 32196678

[9] Vaira LA Salzano G Deiana G De Riu G. Anosmia and ageusia: common findings in COVID-19 patients [published online 4 1, 2020]. Laryngoscope. 10.1002/lary.28692 PMC7228304 32237238

[10] Gane SB Kelly C Hopkins C. Isolated sudden onset anosmia in COVID-19 infection: a novel syndrome [published online 4 2, 2020]? Rhinology. 10.4193/Rhin20.114 32240279

[11] Lechien JR Chiesa-Estomba CM De Siati DR, et al Olfactory and gustatory dysfunctions as a clinical presentation of mild-to-moderate forms of the coronavirus disease (COVID-19): a multicenter European study [published online 4 6, 2020]. Eur Arch Otorhinolaryngol. 10.1007/s00405-020-05965-1 PMC7134551 32253535

[12] Kaye R Chang CW Kazahaya K Brereton J Denneny JC. COVID-19 anosmia reporting tool: initial findings [published online 4 6, 2020]. Otolaryngol Head Neck Surg. 10.1177/0194599820922992 32340555

[13] Moein ST Hashemian SMR Mansourafshar B Khorram-Tousi A Tabarsi P Doty RL. Smell dysfunction: a biomarker for COVID-19 [published online 4 18, 2020]. Int Forum Allergy Rhinol. 10.1002/alr.22587 PMC7262123 32301284

[14] Linder A. Symptom scores as measures of the severity of rhinitis. Clin Allergy. 1988;18:29-37. 3349590 10.1111/j.1365-2222.1988.tb02840.x

[15] Hajian-Tilaki K. Sample size estimation in epidemiologic studies. Caspian J Intern Med. 2011;2:289-298. 24551434 PMC3895825

[16] Arshad Ali S Baloch M Ahmed N Arshad Ali A Iqbal A The outbreak of coronavirus disease 2019 (COVID-19): an emerging global health threat. J Infect Public Health. 2020;13(4):644-646. 32199792 10.1016/j.jiph.2020.02.033 PMC7102664

[17] Omer SB Malani P Del Rio C. The COVID-19 pandemic in the US: a clinical update [published online 4 6, 2020]. JAMA. 10.1001/jama.2020.5788 32250388

[18] Pulla P. Covid-19: India imposes lockdown for 21 days and cases rise. BMJ. 2020;368:m1251. 32217534 10.1136/bmj.m1251

[19] Martinez-Alvarez M Jarde A Usuf E, et al COVID-19 pandemic in West Africa. Lancet Glob Health. 2020;8(5):e631-e632. 32246918 10.1016/S2214-109X(20)30123-6 PMC7186549

[20] Neher RA Dyrdak R Druelle V Hodcroft EB Albert J. Potential impact of seasonal forcing on a SARS-CoV-2 pandemic. Swiss Med Wkly. 2020;150:w20224. 32176808 10.4414/smw.2020.20224

[21] Lai CC Liu YH Wang CY, et al Asymptomatic carrier state, acute respiratory disease, and pneumonia due to severe acute respiratory syndrome coronavirus 2 (SARS-CoV-2): facts and myths [published online 3 4, 2020]. J Microbiol Immunol Infect. 10.1016/j.jmii.2020.02.012 PMC7128959 32173241

[22] Salmon Ceron D Hautefort C Bequignon E Corre A Canoui-Poitrine F Papon JF. Anosmia without nasal obstruction: a pathognomonic sign of COVID-19 infection. In press.

[23] Sungnak W Huang N Bécavin C Berg M, Network HLB. SARS-CoV-2 entry factors are highly expressed in nasal epithelial cells together with innate immune genes [published online ahead of print April 23, 2020]. *Nat Med*. doi:10.1038/s41591-020-0868-6. 10.1038/s41591-020-0868-6 PMC8637938 32327758

[24] Wu C Zheng M. Single-cell RNA expression profiling shows that ACE2, the putative receptor of Wuhan 2019-nCoV, has significant expression in the nasal, mouth, lung and colon tissues, and tends to be co-expressed with HLA-DRB1 in the four tissues. https://www.preprints.org/manuscript/202002.0247/v1. Accessed March 30, 2020.

[25] Zou L Ruan F Huang M, et al SARS-CoV-2 viral load in upper respiratory specimens of infected patients. N Engl J Med. 2020;382:1177-1179. 32074444 10.1056/NEJMc2001737 PMC7121626

[26] McCray PB Jr. Pewe L Wohlford-Lenane C, et al Lethal infection of K18-hACE2 mice infected with severe acute respiratory syndrome coronavirus. J Virol. 2007;81:813-821. 17079315 10.1128/JVI.02012-06 PMC1797474

[27] Netland J Meyerholz DK Moore S Cassell M Perlman S. Severe acute respiratory syndrome coronavirus infection causes neuronal death in the absence of encephalitis in mice transgenic for human ACE2. J Virol. 2008;82:7264-7275. 18495771 10.1128/JVI.00737-08 PMC2493326

[28] Wheeler DL Athmer J Meyerholz DK Perlman S. Murine olfactory bulb interneurons survive infection with a neurotropic coronavirus. J Virol. 2017;91(22):e01099-17. 10.1128/JVI.01099-17 PMC5660484 28835503

[29] Giacomelli A Pezzati L Conti F, et al Self-reported olfactory and taste disorders in SARS-CoV-2 patients: a cross-sectional study [published online 3 26, 2020]. Clin Infect Dis. 10.1093/cid/ciaa330 PMC7184514 32215618

[30] Yan CH Faraji F Prajapati DP Boone CE DeConde AS. Association of chemosensory dysfunction and Covid-19 in patients presenting with influenza-like symptoms [published online 4 12, 2020]. Int Forum Allergy Rhinol. 10.1002/alr.22579 PMC7262089 32279441

[31] Schmier JK Halpern MT. Patient recall and recall bias of health state and health status. Expert Rev Pharmacoecon Outcomes Res. 2004;4:159-163. 19807519 10.1586/14737167.4.2.159

